# Low-glycemic index cookies supplemented with *Cordyceps militaris* substrate: Nutritional values, physicochemical properties, antioxidant activity, bioactive constituents, and bioaccessibility

**DOI:** 10.1016/j.fochx.2025.102494

**Published:** 2025-04-23

**Authors:** Chanh M. Nguyen, Khoa D. Nguyen, Truc N.T. Tran, Tin H. Trang, N.M.N. Ton, V.V.M. Le, T.T.T. Tran

**Affiliations:** a Department of Food Technology, Ho Chi Minh City University of Technology (HCMUT), Ho Chi Minh City, Viet Nam; b Vietnam National University – Ho Chi Minh City (VNU-HCM), Ho Chi Minh City, Viet Nam; c Department of Biochemistry, Faculty of Biology—Biotechnology, University of Science, Ho Chi Minh City, Viet Nam

**Keywords:** *Cordyceps militaris* substrate, Dietary fiber, *In vitro* glycemic index, Antioxidant activity, Bioaccessibility

## Abstract

*Cordyceps militaris* substrate (CMS) is a novel ingredient rich in dietary fibers and bioactive compounds; however, its application in food products remains largely unexplored. This study introduces CMS as a partial wheat flour substitute for developing low-glycemic cookies enriched with dietary fiber and bioactive compounds. Results indicate that CMS supplementation increased fiber and phenolic content by 1.41–2.95 times and 1.30–2.62 times, respectively, compared to unsupplemented cookies. Additionally, carotenoid levels were 15.50–71.34 times higher than those in wheat-based cookies. The simulated digestion system revealed that CMS-supplemented cookies exhibited a low glycemic index (47.9–54.0) while enhancing higher polyphenol and carotenoid release than the controls. Cookies with 15 % CMS supplementation qualified as a “source of fiber”, high-carotenoid, and low-GI food classification while maintaining acceptable sensory attributes. These findings underscore the potential of CMS in food innovation, offering a promising approach to enhancing nutrient intake across populations.

## Highlights

• CMS-added cookies increased dietary fibers 1.41–2.95 times.
• Polyphenols and carotenoids increased 1.30–2.62 times, and 15.50–71.34 times.
• CMS-supplemented cookies lowered GI of 47.9–54.0.
• Antioxidant activity increased 2.1–13.9 times compared with wheat-based cookies.
• Polyphenols and carotenoids bioaccessibility were 35.80–61.50 % and 18.95–10.65 %.

## Graphical abstract

**Figure:**
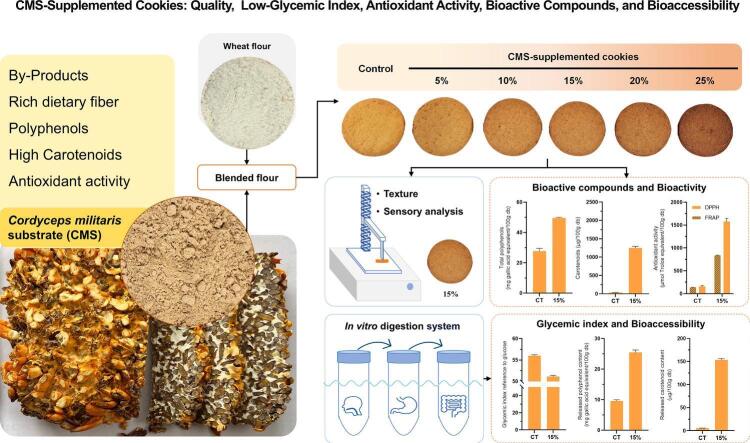

## 1 Introduction

*Cordyceps militaris*, a traditional medicinal mushroom, is rich in protein, polysaccharides, carotenoids, and nucleosides that have been widely applied to provide energy and revitalize bodies (Chan, Barseghyan, Asatiani, & Wasser, 2015). The considerable demand for *C. militaris* in the food supplement and pharmaceutical industries promotes its cultivation. These practices have generated a significant amount of *C. militaris* substrate (CMS), a spent mushroom cultivation medium. According to Ni et al., the annual production of 50–60 tons of dried fruiting bodies generated ten times that amount in rice-based substrate (Ni, Zhou, Li, & Huang, 2009). CMS contains mycelium and a red rice base, which are edible and high in nutrients. According to previous studies, CMS has significant concentrations of minerals Ca, Fe, K, Mg, Na, and Zn, and polyphenols (Jędrejko et al., 2022), and cordycepin (Ni et al., 2009). Polysaccharides extracted from CMS also showed antioxidant and immunomodulation activities (Zeng, Zhang, Zhang, Cui, & Sun, 2015). These findings indicate that CMS has potential as a functional food ingredient. However, research on *C. militaris* primarily focuses on the application of its mycelium, fruiting bodies, or isolated nutraceuticals, while studies on the food applications of CMS remain limited, leaving it an underutilized resource for sustainable food innovation.

Cookies are widely consumed bakery products enjoyed by people of all ages worldwide. However, traditional wheat-based cookies, being high in starch and sugar but low in dietary fiber and bioactive compounds, tend to have a high glycemic index. The glycemic index (GI) quantifies the glucose response triggered by carbohydrate-containing foods relative to that induced by an equivalent carbohydrate intake from a reference food (Ferrer-Mairal et al., 2012; Wolever, Jenkins, Jenkins, & Josse, 1991). Cookies generally have a high glycemic index (approximately 70), making them less suitable for diabetics and individuals managing excess weight or obesity. In contrast, low-glycemic foods offer health benefits, including reduced postprandial hyperglycemia, lower insulin demand, and improved cholesterol levels (Augustin, Franceschi, Jenkins, Kendall, & La Vecchia, 2002).

Previous research suggests that incorporating dietary fiber into cookies can lower their glycemic index (Dartois, Singh, Kaur, & Singh, 2010; Ng, Robert, Ahmad, & Ishak, 2017), potentially making them more suitable for diabetics, individuals with obesity, and weight-conscious consumers. Supplementing cookies with wheat bran, rambutan seed powder, or pitaya peel powder increased their dietary fiber content while simultaneously lowering their glycemic index (GI), classifying them within the medium- or low-GI food group (Mai, Tran, & Le, 2023; Nguyen, Tran, & Le, 2025; Reyes-Pérez, Salazar-García, Romero-Baranzini, Islas-Rubio, & Ramírez-Wong, 2013). In addition to fibers, polyphenols may also block the action of α-amylase and α-glucosidase, thus lowering the GI of foods (Sun & Miao, 2020). Carotenoids and polyphenols may also induce health-promoting effects by preventing free radical-related diseases: diabetes, cancer, and atherosclerosis. Therefore, rich antioxidant ingredients are valuable additions to bakery products (Ngo et al., 2024).

Agricultural by-products, including seeds, pomace, fruit peels, and vegetables, serve as valuable sources of dietary fiber and bioactive compounds. Their incorporation can enrich the nutritional profile and enhance the antioxidant activity of baked goods. Cookies enriched with 11.9 % coffee silverskin powder contained eight times more total fiber, 53.9 % higher total polyphenol content (TPC), and a 74.7 % increase in antioxidant activity compared to the control (Dauber et al., 2024). Similarly, incorporating 12 % turmeric by-products doubled the total fiber content, increased total polyphenol content (TPC) by 6.4 times, and enhanced DPPH scavenging activity by 4.7 times (Le, Nguyen, Ton, & Tran, 2024). Among agricultural by-products, mushrooms present considerable untapped potential for further exploration. However, incorporating by-product powder into cookies alters their physicochemical properties and influences consumer acceptance, necessitating a systematic evaluation for each novel ingredient.

This study examines the potential of CMS as a partial replacement for wheat flour in developing low-glycemic cookies enriched with dietary fiber, bioactive compounds, and antioxidant activity, qualifying them as a “source of fiber” food. The nutritional values, physicochemical properties, and levels of polyphenols, carotenoids, and antioxidant activity in CMS powder were determined and compared with those of wheat flour. CMS powder was then substituted with wheat flour at 5 %, 10 %, 15 %, 20 %, and 25 % to investigate its effects on cookies' nutritional compositions, physicochemical properties, and sensory attributes. Additionally, the glycemic index of the cookies and the bioaccessibility of polyphenols and carotenoids were assessed using an *in vitro* digestion model. The findings may suggest the novel application of CMS as a potential ingredient for bakery goods and provide insight into its impact on cookie attributes.

## 2 Materials and methods

### 2.1 *C. militaris* substrate and cookies ingredients

CMS was supplied by Bioessen CO., Ltd. (Binh Duong, Vietnam) and processed by drying at 60 °C in a hot air dryer until reaching a final moisture content (approximately 2.5 %). The dried CMS was then ground and sifted through a 60-mesh sieve. CMS powder was stored at 5 °C in the dark for subsequent experiments. Other cookie ingredients, including wheat flour, chicken eggs, table salt, isomalt, acesulfame potassium, vanilla powder, baking powder, and butter, were obtained at a local market.

### 2.2 Chemicals

Gallic acid (≥98 %), Folin–Ciocalteu, 2,2-di(4-*tert*-octylphenyl)-1- picrylhydrazyl (DPPH), 2,4,6-tris (2-pyridyl)-*s*-triazine (TPTZ), and dimethyl sulfoxide (DMSO) were bought from Merck KGaA (Germany). Commercial enzymes, including Termamyl® SC, Dextrozyme® GA, Alcalase® 2.5 L, pepsin (1:3000) HI-LRTM, and Pancreatin 4NF/USP, were purchased from Novozymes (Denmark). Other chemicals were of analytical grade and purchased from Merck KGaA (Germany) unless otherwise mentioned.

### 2.3 Cookie preparation

Cookies were prepared with slight modifications according to the described method and formula (Nguyen et al., 2025). Briefly, eggs (23.3 g) were whipped with an electric mixer at 200 rpm for 4 min. Other ingredients, including 0.33 g of table salt, 0.09 g of acesulfame potassium, and 23.3 g of isomalt, were prepared with 6.5 g of water and mixed with whipped eggs at 100 rpm for 1 min. Next, 35 g of butter was incorporated and blended using a hand mixer (HR1456, Philips Co., Guangdong, China) at 200 rpm for approximately 4 min. Vanilla and baking powder, 0.3 and 0.8 g, respectively, were added to the mixture and mixed for 1 min. The obtained mixture was combined with 75 g of blended flour, comprising wheat flour and CMS powder at ratios of 5, 10, 15, 20, and 25 g per 100 g of blended flour. Control cookies were prepared using 100 % wheat flour. The mixture was then blended at 200 rpm for 3 min using a stand mixer (SM8005, Ichiban Ltd., Tokyo, Japan). The cookie dough was then incubated for 30 min before being rolled into a 3-mm sheet and shaped into round pieces using a 45 mm diameter mold. The oven was preheated at 175 °C for 15 min, after which the cookies were baked at 175 °C for 10 min, followed by an additional 5 min at 150 °C in an oven (GL-1126, Gali Co., Ho Chi Minh City, Vietnam). The baked cookies were then cooled to 25 °C at room temperature with an air humidity of 75–80 %. Finally, the cookies were stored in sealed plastic bags in a cool, dry place for further analysis.

### 2.4 Analytical methods

#### 2.4.1 Nutritional composition

The nutritional composition of wheat flour, CMS powder, and cookies was measured according to the Association of Official Analytical Chemists (International, 2000). Moisture was determined using an MX-50 infrared moisture analyzer (A&D Co., Japan). Protein was measured using the Kjeldahl method performed according to AOAC 984.13. Lipid was determined using AOAC 960.39. Ash was determined according to AOAC 930.30, and starch was measured using AOAC 996.11. Total dietary fiber (TDF), insoluble dietary fiber (IDF), and soluble dietary fiber (SDF) were determined following AOAC 991.43, 991.42, and 993.19 methods, respectively. The energy value of the cookies was estimated from the relative content of the protein, fat, and carbohydrates, using the Atwater general factors of 4.0, 9.0, and 4.0 kcal/g, respectively (Capuano, Oliviero, Fogliano, & Pellegrini, 2018). Free fatty acid (FFA) was determined according to ISO 660:2009, and the peroxide value analysis followed ISO 3960:2017 standards. The fatty acid profile was assessed by the AOAC 996.06 method, using gas chromatography–mass spectrometry (GC–MS) (Agilent 7890B/MSD 5977 A, Agilent Technology, Santa Clara, CA, USA) and an HP 88 GC column (30 m × 250 μm × 0.5 μm) (Agilent Technology, Santa Clara, CA, USA) (Nguyen et al., 2025).

#### 2.4.2 Determination of total polyphenol content

Total polyphenol content was measured according to the previous method (Le et al., 2024). Diluted samples were reacted with a 10-fold diluted Folin–Ciocalteu solution in the presence of 10 % Na_2_CO_3_. After incubating in the dark for 2 h, the absorbance of samples was measured at 760 nm using a UV–Vis spectrophotometer (UV 2505, Labomed, USA). Gallic acid was used as an analytical standard with the calibration curve, y = 82.916*OD - 0.0546 (R^2^ = 0.9995). TPC of samples was expressed in milligrams of gallic acid equivalent (GAE) per 100 g of dry basis (mg GAE/100 g db).

#### 2.4.3 Determination of carotenoid content

Total carotenoid content was determined using the method reported by Lichtenthaler, H.K. et al. (Lichtenthaler & Buschmann, 2001) with slight modifications. Samples were extracted with 95 % ethanol in the dark at room temperature for 24 h. The extract was collected by centrifuging at 6000 rpm for 15 min, and the absorbance was measured at three wavelengths of 470, 645, and 662 nm.

The carotenoid content was calculated using the following formulas:

$$C_{a}=13.36\times A_{662}-5.19\times A_{645}$$

$$C_{b}=27.43\times A_{645}-8.12\times A_{662}$$

$$C_{\text{carotenoids}}=\left(1000\times A_{470}-2.13\times C_{a}-97.64\times C_{b}\right)/209$$

Carotenoid content was expressed in micrograms per 100 g of dry basis (μg/100 g db).

#### 2.4.4 Antioxidant activity

Antioxidant activity was determined through DPPH and ferric-reducing antioxidant power (FRAP) assays (Le et al., 2024). For the former, 0.1 mL of diluted samples was added to 3.9 mL of DPPH solution (20 mg/L DPPH in MeOH). The reaction occurred in the dark at room temperature for 30 min, and the absorbance was recorded at 515 nm against MeOH using a UV–Vis spectrophotometer (UV 2505, Labomed, USA). The percentage of DPPH scavenging was determined using the following formula:

$$\text{Scavenging activity}\;\left(\%\right)=\left(A_{\mathrm{NC}}-A_{t}\right)/A_{\mathrm{NC}}\;x\;100$$

Where A_NC_ denoted the absorbance of the negative control, and A_t_ indicated the absorbance of the tested samples. Trolox (0–1000 μM) was used as an analytical standard. The calibration curve was fitted based on the correlation of scavenging activity and the concentration of Trolox, y = 24.675*OD – 9.5621 (R^2^ = 0.996). The scavenging activity of samples was expressed in micromoles of Trolox equivalent per gram of dry basis (μmol TE/g db). As for the FRAP assay, 3.4 mL of the FRAP working solution was mixed with 0.6 mL of the diluted extract. The reaction mixture occurred in the dark at 37 °C for 5 min, the absorbance was measured at 593 nm. The reducing power of samples was expressed in μmol TE/g db.

#### 2.4.5 Physical properties

The cookie's diameter, thickness, and spread ratio, were evaluated following the previously reported procedure (Le et al., 2024). The diameters (D, mm) of six cookies arranged in a horizontal row were measured using a digital vernier caliper. Each cookie was measured for two readings after rotating 90°. Thickness (T) was determined by measuring the height of stacks of six cookies and calculating the average thickness. The spreading factor (SF) was calculated by the ratio between the diameter and thickness (SF = D/T). The hardness and fracturability of cookies were determined using three breaking points measurements performed with a texture profile analyzer (TA-XT Plus, Stable Micro System, UK) and HDP/3 PB probe (Šarić et al., 2019). Water and oil holding capacities were determined according to the previously described method (Fernández-López et al., 2009). Samples (1 g) and 10 mL of water or oil were put into a centrifuge tube. The tube was vortexed for 30 s and kept at room temperature for 2 h. The mixture was centrifuged at 3000 rpm for 10 min, and the supernatant was removed. The difference in weight between the initial sample and the sediment was used to calculate the absorbed amount of water or oil. Results were presented in grams of water or oil per gram of the dry basis sample. The color of samples was measured using a colorimeter CR400 (Konica Minolta, Japan) in the CIELAB color space, where L* (lightness), a* (redness-greenness), and b* (yellowness-blueness) values were recorded. The total color difference of the samples (∆E) was calculated by the formula:

$$∆E=\sqrt{{\left({a_{0}}^{*}-a^{*}\right)}^{2}+{\left({b_{0}}^{*}-b^{*}\right)}^{2}+{\left({L_{0}}^{*}-L^{*}\right)}^{2}\;}$$

where a_0_*, b_0_*, and L_0_* values of control sample, and a*, b*, and L* values of tested samples.

#### 2.4.6 Sensory evaluation

The overall acceptance of cookie samples was assessed using a nine-point hedonic scale, ranging from 1 (extremely dislike) to 9 (extremely like). Sensory evaluation was conducted by 60 healthy, untrained panelists aged 18 to 25 years, selected from Food Technology students of Ho Chi Minh City University of Technology (HCMUT). Each cookie sample was labeled with a unique three-digit code, and five samples were presented simultaneously in a randomized order. Water was provided for palate cleansing between the samples (Nguyen et al., 2025). This sensory assessment did not require formal ethical approval, given the low risk posed to healthy panelists. However, throughout the study, participants' rights and privacy were safeguarded through appropriate protocols. All volunteers provided informed consent to participate in the sensory evaluation and permitted the use of their responses for research purposes.

#### 2.4.7 *In vitro* glycemic index

The glycemic index of cookies was measured using an *in vitro* simulated method (Goñi, Garcia-Alonso, & Saura-Calixto, 1997). Samples containing an equivalent amount of one gram of carbohydrate were added to 30 mL of water with an adjusted pH of 2.5 by HCl 1 M, followed by the addition of 1 mL of pepsin (2000 U/mL). The simulated gastric phase was kept at 37 °C, with agitation of 100 rpm for 30 min. The pH of simulated gastric fluid was adjusted to 6.5 by NaHCO_3_ 1 M, followed by the addition of 5 mL enzyme pancreatin (100 U/mL) and 0.1 mL glucoamylase (260 U/mL). The intestinal phase was carried out at 37 °C for 2.5 h with agitation of 100 rpm. During the incubation period, an aliquot of the sample was taken at 30, 60, 90, 120, 150, and 180 min for glucose determination using the 3,5-dinitro salicylic acid method. The kinetic concentration of glucose released was determined by the following formula:

$$C=C_{\infty }\left(1-e^{-\mathrm{kt}}\right)$$

Where *C* corresponds to the concentration (g/L) of glucose at the measured time (t), C_∞_ corresponds to the concentration at equilibrium (180 min), and k is the kinetic constant. The area under the curve of glucose response was determined and the hydrolysis index (HI) was calculated as follows:

$$\mathrm{HI}=\frac{{\mathrm{AUC}}_{s}}{{\mathrm{AUC}}_{r}}\times 100$$

Where AUCs is the area under the curve of glucose released from cookie samples and AUCr is that of white bread.

The expected glycemic index (pGI) was calculated using the following formula:

pGI=39.71+0.549HI

The glycemic index was converted into glucose reference by multiplying pGI with 0.71.

#### 2.4.8 Bioaccessibility of polyphenols and carotenoids

The bioaccessibilities of polyphenols and carotenoids were determined based on published studies (Lucas-González et al., 2021; Petry & Mercadante, 2017). The intestinal fluid of the sample was taken, and the polyphenols and carotenoid content were determined. The bioaccessibility was calculated based on the following formula:

$$\mathrm{Bioaccessibility}=\frac{A_{1}}{A_{2}}\times 100$$

Where A_1_ corresponds to the polyphenol or carotenoid content of samples at the intestinal phase, A_2_ corresponds to the bioactive compounds in undigested cookies.

and A_2_.

### 2.5 Statistical analysis

All experimental values were conducted in triplicate and data was presented in means ± SD. The statistical comparison was performed using a one-way Analysis of Variance (Statgraphics Centurion XV.I.). The significance of the difference was conducted using Multiple range tests (*p* < 0.05) using MODDE 13.0 software.

## 3 Results and discussion

### 3.1 Nutritional compositions, physicochemical properties, bioactive levels, and antioxidant activity of *C. militaris* substrate powder

The nutritional value, physicochemical properties, bioactive compounds, and antioxidant activity of CMS were determined and compared with those of wheat flour. The results show that the nutrients in CMS powder were more abundant than wheat flour for most nutritional compositions except starch and carbohydrates (Table 1). The protein, lipid, and ash contents in CMS were approximately 1.5, 2.6, and 3.0 times higher than those in wheat flour. The prominent ash content indicates the high levels of minerals in CMS. This was consistent with the previous study by Jedrejko et al., showing that the harvested CMS after fruiting body cultivation contained high levels of Ca, Fe, K, Mg, Na, and Zn but without Cu and Mn (Jędrejko et al., 2022). These are such important minerals in the human diet. Noticeably, the total dietary fiber (TDF), insoluble dietary fiber (IDF), and soluble dietary fiber (SDF) were 8.39, 10.82, and 5.46 times greater than those in wheat flour. In addition, while the IDF-to-SDF ratio (IDF/SDF) of wheat flour was 1.2, CMS powder exhibited a ratio of 2.4, aligning more closely with the recommended 2:1 proportion in food (Khanpit, Tajane, & Mandavgane, 2021). The prominent levels and a good proportion of IDF/SDF indicate that CMS is a valuable source of dietary fiber for functional cookies.

**Table 1:** Comparative analysis of the nutritional composition, physicochemical properties, bioactive content, and antioxidant activity of *C. militaris* substrate powder and wheat flour.

Features	Component	Unit	Wheat four	*C. militaris* substrate powder
Nutritional compositions	Protein	g/100 g db	9.68 ± 0.23^a^	14.22 ± 0.20ᵇ
	Lipid	g/100 g db	2.83 ± 0.07^a^	7.32 ± 0.11^b^
	Ash	g/100 g db	0.58 ± 0.04^a^	1.75 ± 0.03^b^
	Carbohydrates	g/100 g db	86.34 ± 0.47^b^	79.64 ± 0.10^a^
	Starch	g/100 g db	80.00 ± 0.26^b^	42.04 ± 0.11^a^
	TDF	g/100 g db	2.12 ± 0.13^a^	17.78 ± 0.72ᵇ
	IDF	g/100 g db	1.17 ± 0.13^a^	12.66 ± 0.45ᵇ
	SDF	g/100 g db	0.96 ± 0.03^a^	5.24 ± 0.28^b^
Physico-chemical properties	L*	–	93.03 ± 0.08^b^	69.61 ± 0.14^a^
	a*	–	0.31 ± 0.03^a^	6.33 ± 0.09^b^
	b*	–	6.70 ± 0.08^a^	16.83 ± 0.14^b^
	ΔE	–	–	26.22 ± 0.12
	Oil holding capacity	g oil/g db	1.07 ± 0.08ᵃ	1.31 ± 0.01ᵇ
	Water holding capacity	g water/g db	0.90 ± 0.04^a^	3.87 ± 0.03^b^
Bioactive levels	Total phenolics	mg GAE/100 g db	87.40 ± 4.01^a^	469.17 ± 3.90ᵇ
	Carotenoids	μg/100 g db	–	7958.22 ± 481.00
Antioxidant capacity	DPPH	μmol TE/100 g db	242.81 ± 22.59^a^	12,775.13 ± 133.43ᵇ
	FRAP	μmol TE/100 g db	203.46 ± 8.52^a^	6226.45 ± 30.96ᵇ

Data are presented as mean ± standard deviation (n = 3). Means with different superscript letters in each row indicate significant differences (p < 0.05). Abbreviations: db, dry basis; TDF, total dietary fiber; IDF, insoluble dietary fiber; SDF, soluble dietary fiber; DPPH, DPPH radical-scavenging assay; FRAP, Ferric Reducing Antioxidant Power; GAE, gallic acid equivalent; TE: Trolox equivalent.

As for physicochemical properties, the color, oil-holding capacity, and water-holding capacity of CMS powder were determined and compared with those of wheat flour. The two powders exhibited distinct color differences, with the lightness (L* value) of CMS powder being 25.5 % lower than that of wheat flour, while its redness (a*) and yellowness (b*) were markedly greater than those of the control. These values show that the color of CMS powder was darker and more intense in red and yellow hues compared with wheat flour. The total color difference (ΔE) was 26.22 ± 0.12, considerably greater than 5, indicating that the difference is clear and can be easily recognizable by the naked eye. The great difference in color between these two materials could be due to the high pigment content in CMS powder. In addition, the water-holding capacity of CMS was 3.87 g water/g db, and the oil-holding capacity was 1.31 g oil/g db, 4.30 and 1.22 times as much as those of wheat flour, respectively. The capacity to hold water or oil could be attributed to several factors, such as the fiber composition, structure of spores or capillaries, and functional groups that can interact with water or lipid molecules (Foschia, Peressini, Sensidoni, & Brennan, 2013). These results suggest that the incorporation of CMS powder into cookies should alter their physicochemical features.

Not only the nutritional values and physicochemical properties, but CMS powder also showed marked differences in bioactive compounds and antioxidant activity compared with wheat flour.

In addition to its nutritional values and physicochemical properties, CMS powder showed significant differences in bioactive compounds and antioxidant activity compared with wheat flour. Its total polyphenol content was 469.17 ± 3.90 mg GAE/100 g db, 5.4 times as much as that of wheat flour. Furthermore, the carotenoid concentration—the major pigment causing the intense yellowness and redness of CMS powder—was 7958.22 ± 481.00 μg/100 g db, whereas no carotenoids were detected in wheat flour. The scavenging ability against DPPH free radicals and ferric-reducing power of CMS powder were 52.61 and 30.60 times greater than those of wheat flour, respectively. The antioxidant actions of CMS powder corresponded to its higher TPC and carotenoid content.

### 3.2 The nutritional composition of CMS-supplemented cookies

The incorporation of CMS powder into cookies changed their nutritional values. Increasing the CMS supplementation ratio from 0 % (control) to 25 % resulted in a protein content elevation from 9.39 % to 9.63 % and a lipid content increase from 21.26 % to 24.39 %, while carbohydrates and starch decreased from 67.87 % to 64.38 % and from 52.46 % to 42.33 %, respectively (Table 2). Dietary fiber content in cookies increased with CMS supplementation, from 1.48 to 4.37 g/100 g db. Notably, at a 15 % supplementation level, TDF reached 3.22 ± 0.17 g/100 g db, qualifying the cookies as a “source of fiber” (TDF at least 3 g/100 g db) according to European Regulation (EC) No 1924/2006 on nutrition and health claims made on foods (Zicari, Carraro, & Bonetta, 2007). The insoluble-to-soluble dietary fiber (IDF/SDF) ratio also increased from 1.60 to 2.39 as CMS supplementation increased from 0 % to 25 %. Previous research suggested that an IDF/SDF ratio of roughly 2.0 would optimize the health benefits of dietary fibers (Khanpit et al., 2021). The energy value of control cookies was approximately 494.41 Kcal/100 g db, while CMS supplementation led to a slight increase, ranging from 495.81 to 498.04 Kcal/100 g db (*P* < 0.05). Notably, the energy content remained relatively stable despite CMS addition, which could be attributed to the high protein and lipid content counteracted by low starch and carbohydrate levels. In addition, free fatty acid (FFA) increased in relation to CMS supplementation, from 0.04 % in control cookies to 0.08–0.16 % in CMS-supplemented cookies (Table 2). This slight increase corresponds to the small rise in lipid and moisture content of the samples. Despite a modest increase in free fatty acid content, peroxide values significantly declined, from 2.25 meqO₂/kg in control cookies to 1.82–1.25 meqO₂/kg in CMS-supplemented cookies, indicating reduced lipid oxidation. This phenomenon can be attributed to two key factors. First, the high oil-holding capacity of CMS powder (Table 1) enables it to interact with and retain lipid molecules within its structure, thereby reducing oxidation during baking. Second, the elevated levels of natural antioxidant compounds, including total phenolic and carotenoid content (Table 1), provide enhanced oxidative protection, mitigating lipid degradation. To evaluate the oxidative stability of cookies, the fatty acid profiles of control and 15 % CMS-supplemented cookies were analyzed and compared (Table S1). The results revealed a similar composition between both groups, with the predominant fatty acids including palmitic acid (8.670–8.880 %), lauric acid (1.394–1.460 %), and stearic acid (1.245–1.270 %) among saturated fatty acids, as well as oleic acid (7.650–7.970 %) and linoleic acid (2.620–2.760 %) among unsaturated fatty acids. These results align with previous findings, indicating that palmitic acid, oleic acid, and linoleic acid are the predominant fatty acids commonly present in cookies (Kaur, Choudhary, & Sharma, 2023).

**Table 2:** The nutritional composition and peroxide value of CMS-supplemented cookies.

Composition (g/100 g db)	Control	CMS-supplemented cookies				
		5 %	10 %	15 %	20 %	25 %
Moisture	2.40 ± 0.03ᵃ	2.65 ± 0.07ᵇ	2.98 ± 0.03ᶜ	3.18 ± 0.04ᵈ	3.39 ± 0.03ᵉ	3.53 ± 0.03ᶠ
Protein	9.39 ± 0.01ᵃ	9.43 ± 0.01ᵇ	9.48 ± 0.02^c^	9.53 ± 0.01^d^	9.58 ± 0.01^e^	9.63 ± 0.01^f^
Lipid	21.26 ± 0.34ᵃ	22.05 ± 0.41ᵇ	22.67 ± 0.13^c^	23.23 ± 0.09^d^	23.73 ± 0.22^e^	24.39 ± 0.33^f^
Ash	1.49 ± 0.01^a^	1.52 ± 0.02^b^	1.55 ± 0.01^c^	1.58 ± 0.01^d^	1.61 ± 0.01^e^	1.65 ± 0.01^f^
Carbohydrates	67.87 ± 0.35^f^	67.00 ± 0.42^e^	66.29 ± 0.12^d^	65.66 ± 0.09^c^	65.07 ± 0.20^b^	64.38 ± 0.32^a^
Starch	52.46 ± 0.04^f^	49.98 ± 0.16^e^	48.93 ± 1.27^d^	45.81 ± 0.39^c^	43.53 ± 0.24^b^	42.33 ± 0.45^a^
Energy (Kcal)	494.41 ± 1.32ᵃ	495.81 ± 2.18ᵃᵇ	496.63 ± 0.18ᵃᵇ	496.97 ± 1.05ᵇ	496.47 ± 1.11ᵃᵇ	498.04 ± 1.82ᵇ
TDF	1.48 ± 0.11^a^	2.09 ± 0.04^b^	2.63 ± 0.11^c^	3.22 ± 0.17^d^	3.93 ± 0.10^e^	4.37 ± 0.03^f^
IDF	0.91 ± 0.11ᵃ	1.32 ± 0.02ᵇ	1.74 ± 0.04^c^	2.21 ± 0.12^d^	2.75 ± 0.11^e^	3.08 ± 0.03^f^
SDF	0.57 ± 0.03ᵃ	0.77 ± 0.06ᵇ	0.89 ± 0.07^c^	1.01 ± 0.05^d^	1.18 ± 0.07^e^	1.29 ± 0.03^f^
IDF/SDF	1.60 ± 0.22ᵃ	1.72 ± 0.17ᵇ	1.96 ± 0.12^c^	2.19 ± 0.02^d^	2.34 ± 0.20^e^	2.39 ± 0.07^f^
Free fatty acids	0.04 ± 0.00ᵃ	0.08 ± 0.00ᵇ	0.11 ± 0.01ᶜ	0.12 ± 0.01ᵈ	0.13 ± 0.01ᵉ	0.16 ± 0.01ᶠ
Peroxide value (meqO_2_/ Kg)	2.25 ± 0.04ᶠ	1.82 ± 0.02ᵉ	1.61 ± 0.02ᵈ	1.42 ± 0.03ᶜ	1.32 ± 0.03ᵇ	1.25 ± 0.02ᵃ

Data are presented as mean ± standard deviation (*n* = 3). Means with different superscript letters in each row indicate significant differences (*p* < 0.05). Abbreviations: CT: control cookies without CMS supplementaton; 5 %, 10 %, 15 %, 15 %, 20 %, 25 %: cookies supplemented with 5 %, 10 %, 15 %, 15 %, 20 %, and 25 % CMS; CMS: *C. militaris* substrate; db, dry basis; TDF, total dietary fiber; IDF, insoluble dietary fiber; SDF, soluble dietary fiber; meq O2/kg, milliequivalents of oxygen per kilogram.

### 3.3 Total polyphenol, carotenoid content, and antioxidant activity of CMS-supplemented cookies

**Fig. 1:**
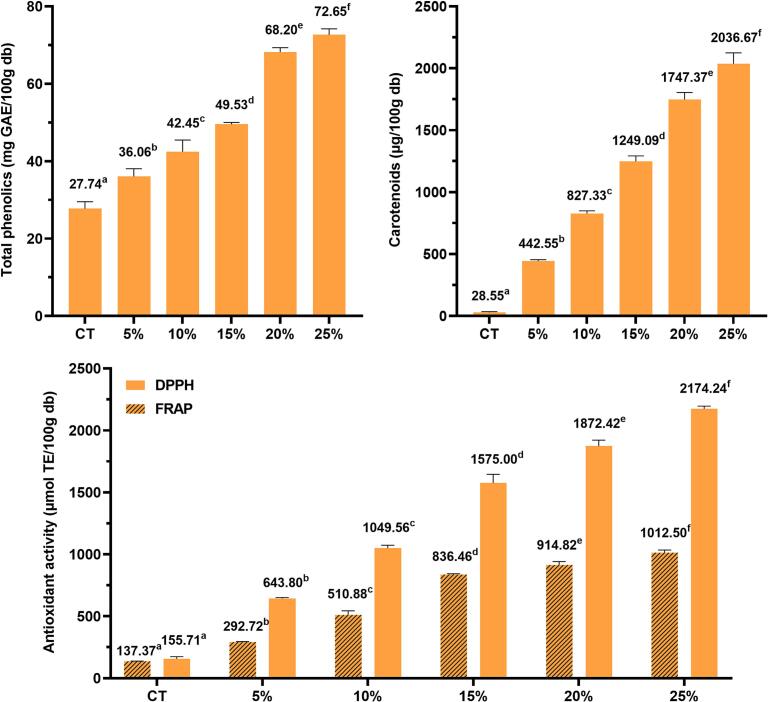
Total polyphenolics, carotenoids, and antioxidant activities of CMS-supplemented cookies. Abbreviations: CT: control cookies without CMS supplementaton; 5 %, 10 %, 15 %, 15 %, 20 %, 25 %: cookies supplemented with 5 %, 10 %, 15 %, 15 %, 20 %, and 25 % CMS; CMS: *C. militaris* substrate; GAE: gallic acid equivalent; TE: Trolox equivalent.db: dry basis; Values with different superscript letters show the significant difference among columns or assays (p < 0.05).

In addition to nutritional values, cookies' total polyphenol and carotenoid content increased significantly. As for the total polyphenol compounds, this bioactive content markedly increased from 27.74 to 72.65 mg GAE/100 g db (Fig. 1A). Regarding carotenoids, the substances escalated 15.50–71.34 times as much as the control (Fig. 1B). According to Britton et al.'s classification, control cookies contained very low carotenoid levels (0–0.1 mg/100 g). With 5 % CMS supplementation, cookies fell into the moderate carotenoid food group (0.1–0.5 mg/100 g), while those supplemented with 10–20 % CMS were classified as high-carotenoid foods (0.5–2 mg/100 g). Cookies with 25 % CMS supplementation belonged to the very high carotenoid food group (>2 mg/100 g), similar to naturally carotenoid-rich foods such as tomatoes, carrots, and apricots (Britton & Khachik, 2009). The increase in these bioactive substances was positively proportional to the supplemented ratio of CMS powder. Moreover, antioxidant activity improved substantially. The DPPH scavenging ability and ferric reducing power of cookies increased from 155.71 to 2174.24 μmol TE/100 g and from 137.37 to 1012.50 μmol TE/100 g, respectively (Fig. 1C). Several factors could contribute to the enhanced antioxidant activity in CMS-supplemented cookies. Primarily, the elevated levels of antioxidant compounds—total polyphenols (TPC) and carotenoids—directly enhance radical scavenging and ion-reducing capacity. Additionally, interactions between dietary fiber and polyphenols may facilitate the formation of esterified compounds, resulting in greater antioxidant activity compared to individual components. According to previous studies, dietary fiber can interact with bioactive compounds, such as phenolics forming antioxidant dietary fiber and exhibiting antioxidant activity (Dauber et al., 2024).

### 3.4 Physicochemical properties and sensory evaluations of CMS-supplemented cookies

**Fig. 2:**
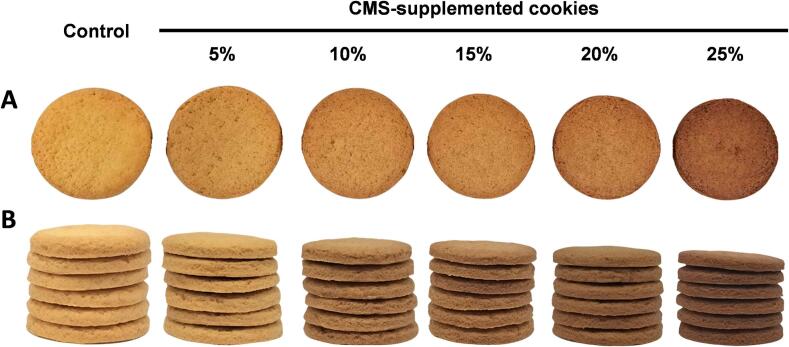
Color (A) and thickness (B) of CMS-supplemented cookies. Abbreviations: CT: Control cookies without CMS supplementation; 5 %, 10 %, 15 %, 15 %, 20 %, 25 %: Cookies supplemented with 5 %, 10 %, 15 %, 15 %, 20 %, and 25 % CMS; CMS: *C. militaris* Substrate

The incorporation of CMS powder into cookies induced slight dimensional changes, with increasing supplementation gradually reducing thickness from 26.72 mm to 25.95 mm and diameter from 3.73 mm to 3.51 mm (Table 3 & Fig. 2A). This could be attributed to reduced gluten protein and decreased free water content in cookie dough, as both factors influence dough structure and strength. A weakened gluten network and high hydration contain restrict dough expansion during baking, ultimately resulting in cookie shrinkage (Barak, Mudgil, & Singh Khatkar, 2013). This result was consistent with a previous study, which reported a reduction in cookie diameter and thickness following supplementation with varying levels of turmeric powder (Le et al., 2024). In addition to diameter and thickness, the spreading factor (SF)—defined as the diameter-to-thickness ratio— is a key indicator of cookie quality. Because thickness decreased to a greater extent than diameter, the SF increased slightly from 7.16 to 7.39 (Table 3). The SF was similar to a previous record on cookies supplemented with passion fruit epicarp flour (Ning et al., 2021). Additionally, the color of the CMS-supplemented cookies differed significantly from their wheat-based counterparts. The lightness and yellowness decreased from 68.46 to 48.48 and from 29.23 to 16.60, respectively, while redness increased proportionally with CMS supplementation. At 25 % CMS supplementation, redness values were two units greater than the control (Table 3). The total color difference between supplemented and control cookies ranged from 6.13 to 23.75, significantly greater than the threshold of five, indicating visually perceptible differences in coloration (Fig. 2B). The variation in cookie color may be due to the difference in the coloration of raw materials (Table 1). In addition, the Maillard reaction may contribute to color development, as the higher protein content in CMS powder than in wheat flour may facilitate more pronounced interaction between proteins and reducing sugars during baking, leading to a darker cookie appearance.

**Table 3:** Physicochemical properties and sensory evaluation of CMS-supplemented cookies.

Features	Unit	Control	CMS-supplemented cookies				
			5 %	10 %	15 %	20 %	25 %
Diameter	mm	26.72 ± 0.08^f^	26.56 ± 0.03^e^	26.36 ± 0.05^d^	26.23 ± 0.01^c^	26.12 ± 0.04^b^	25.95 ± 0.09^a^
Thickness	mm	3.73 ± 0.02^f^	3.68 ± 0.01^e^	3.65 ± 0.01^d^	3.62 ± 0.01^c^	3.59 ± 0.02^b^	3.51 ± 0.01^a^
Spreading factor (D/T)	–	7.16 ± 0.02^a^	7.22 ± 0.02ᵇ	7.23 ± 0.02ᶜ	7.24 ± 0.01ᵈ	7.28 ± 0.03^e^	7.39 ± 0.03^f^
Hardness	g	490.06 ± 0.62^a^	572.45 ± 7.78ᵇ	659.65 ± 8.41ᶜ	744.48 ± 12.48ᵈ	866.56 ± 19.74^e^	992.88 ± 82.18^f^
Fracturability	mm	0.22 ± 0.02^a^	0.26 ± 0.01ᵇ	0.28 ± 0.01ᶜ	0.31 ± 0.01ᵈ	0.34 ± 0.01^e^	0.39 ± 0.03^f^
L*	–	68.46 ± 0.40^f^	63.62 ± 0.26^e^	56.96 ± 0.08^d^	54.53 ± 0.13^c^	52.36 ± 0.26^b^	48.48 ± 0.25^a^
a*	–	8.40 ± 0.15^a^	9.29 ± 0.07ᵇ	9.59 ± 0.03ᶜ	10.02 ± 0.04ᵈ	10.26 ± 0.02^e^	10.55 ± 0.02^f^
b*	–	29.23 ± 0.39^f^	25.58 ± 0.03^e^	22.31 ± 0.09^d^	20.28 ± 0.38^c^	18.60 ± 0.07^b^	16.60 ± 0.54^a^
ΔE	–	–	6.13 ± 0.18^a^	13.48 ± 0.31^b^	16.64 ± 0.26^c^	19.39 ± 0.36^d^	23.75 ± 0.11^e^
Overall preference**	–	6.75 ± 0.95^a^	6.85 ± 1.20^a^	6.87 ± 1.28^a^	7.00 ± 1.10^a^	6.25 ± 1.30^b^	5.45 ± 1.27^c^

Data are presented as mean ± standard deviation (*n* = 3). Means with different superscript letters in each row indicate significant differences (*p* < 0.05); Abbreviations: CT: control cookies without CMS supplementaton; 5 %, 10 %, 15 %, 15 %, 20 %, 25 %: cookies supplemented with 5 %, 10 %, 15 %, 15 %, 20 %, and 25 % CMS; CMS: *C. militaris* substrate;**Nine-point hedonic scale:1 – Extremely dislike; 2 – Moderately dislike; 3 – Regularly dislike; 4 – Slightly dislike; 5 – Neither like nor dislike; 6 – Slightly like; 7 – Regularly like; 8 – Moderately like; 9 – Extremely like.

CMS supplementation increased cookie hardness from 490 to 993 g, which could be attributed to the cumulative content of the total dietary fiber (Mancebo, Picón, & Gómez, 2015). These findings were consistent with previous reports, recording that the addition of rich-fiber ingredients increased cookies' hardness. The hardness of high-fiber, high-protein cookies supplemented with spirulina powder, sorghum flour, and guar gum ranged from 1545.14 to 2298.14 g (Singh, Singh, Jha, Rasane, & Gautam, 2015), whereas cookies supplemented with 3–12 % turmeric by-products exhibited hardness values between 1413 and 1775 g (Le et al., 2024). This effect could be attributed to the interference of fiber with gluten-protein interactions, leading to a reduced-strength gluten network. In addition, the water-holding capacity of dietary fiber in CMS could binding more water within fiber matrix, thus limiting free water availability in the dough and making cookies harder during the baking process (Nićetin et al., 2024).

Along with hardness, the addition of CMS powder increased the fracturability of cookies. Fracturability is a parameter describing how fragile cookies can break. The increase in the fracturability means cookies are easier to break by external force. The fracturability of cookies complemented with 25 % of CMS was 1.77 times as much as the control (Table 3). Comparable results were recorded in an earlier study; Parul Singh et al. showed that adding spirulina powder, sorghum flour, and guar gum increased the fracturability of cookies (Singh et al., 2015). These findings suggest that dietary fibers reduced the resistance of cookies to fracture tests (Wang, Li, & Gao, 2014).

The sensory evaluation of CMS-supplemented cookies is presented in Table 3. The overall preference scores for cookies supplemented with 5–15 % CMS ranged from 6.85 to 7.00, showing no significant differences from wheat-based cookies. However, at 20–25 % CMS supplementation, overall acceptability declined, with sensory scores decreasing to 5.45–6.25. This reduction may be related to the changes in the color and texture of cookies (Table 3), as 20–25 % CMS supplementation rendered cookies darker, harder, and more fragile, thus decreasing overall preferences. In addition, the bitter taste of the cookies could be attributed to the low sensory scores in the cookies with the higher substitution of wheat flour.

### 3.5 *In vitro* glycemic index of CMS-supplemented cookies

**Fig. 3:**
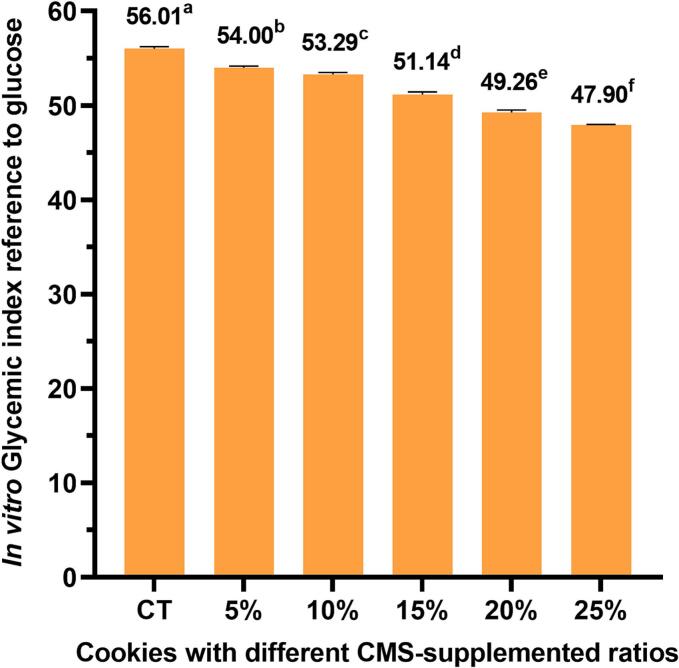
The *in vitro* glycemic index of CMS-supplemented cookies. Abbreviations: CT: control cookies without CMS supplementaton; 5 %, 10 %, 15 %, 15 %, 20 %, 25 %: cookies supplemented with 5 %, 10 %, 15 %, 15 %, 20 %, and 25 % CMS; CMS: *C. militaris* substrate; Values with different superscript letters show the significant difference among columns or assays (p < 0.05).

The glycemic index quantifies the postprandial blood glucose response relative to an equivalent carbohydrate intake of white bread or glucose (Wolever et al., 1991). A higher rate of carbohydrate absorption induces an elevated glycemic index. Low-GI foods are widely recognized for their health benefits, including the prevention or delayed onset of insulin resistance-related diseases (Brand-Miller, 2004) and chronic conditions associated with certain cancers, such as coronary heart disease (Augustin et al., 2002). In this study, the GI of CMS-supplemented cookies was assessed using an *in vitro* assay, which simulates the upper gastrointestinal tract during carbohydrate digestion, which showed a good correlation to the *in vivo* GI values (Goñi et al., 1997). The GI of cookies is presented in Fig. 3. It is noted that the control cookies had a GI of 56.04, classifying them within the medium-GI food group (GI = 56–69), which was lower than conventional cookies with a typically high GI of 70–80. This reduction was attributed to the replacement of sugar with isomalt and acesulfame potassium in the cookie recipe. Incorporation of CMS further lowered the GI of control cookies to 47.90–54.00 (Fig. 3), placing them in the low-GI food group (GI < 55) (*P* < 0.05). The reduction in GI could be attributed to an increase in cookies' dietary fiber. SDF has been reported to lower the probability of α-amylase-mediated carbohydrate breakdown by increasing the viscosity of digested fluid (Dartois et al., 2010). These results were consistent with previous research. Cookies supplemented with 30 % extruded wheat bran contained twice the total dietary fiber, resulting in a medium GI of 68.54 (Reyes-Pérez et al., 2013). Similarly, cookies supplemented with 40 % rambutan seed powder contained 2.3 times more dietary fiber and showed a 30 % reduction in GI compared with controls (Nguyen et al., 2025). Incorporation of 5–25 % pitaya peel powder into cookies also increased fiber 7.1 times, producing low-GI cookies with values ranging from 45.7 to 51.9 (Mai et al., 2023). In addition, β-glucan—the primary component of fungal dietary fiber—slowed down the rate of glucose absorption by forming a separating layer between the enzyme and intestine (Ng et al., 2017). The increase in polyphenol content may also inhibit the activity of α-amylase and α-glucosidase enzymes, resulting in reduced GI (Sun & Miao, 2020).

### 3.6 Polyphenols and carotenoids bioaccessibility of CMS-supplemented cookies

Bioaccessibility of polyphenol and carotenoid compounds refers to the proportion of these natural compounds released from CMS-supplemented cookies during *in vitro* digestion. Increasing CMS supplementation enhanced the availability of polyphenol and carotenoid contents, with concentrations increasing from 9.57 to 45.27 mg GAE/100 g db and from 5.41 to 216.83 μg/100 g db, respectively (Table 4). The bioaccessibility of polyphenols increased proportionally, from 35.80 % to 61.50 %, aligning with findings on low-fat functional cookies enriched with wheat germ flour and coffee silverskin (Büyük & Dulger Altiner, 2024). However, the bioaccessibility of carotenoids decreased from 18.95 % to 10.65 %, although the available content of carotenoids increased. This discrepancy occurred because the increase in carotenoid levels in CMS-supplemented cookies was substantially greater than the proportion released during digestion. The bioaccessibility of carotenoids was consistent with a previous study, which reported intestinal release ratios ranging from 10 % to 30 % (Van Loo-Bouwman et al., 2010). The reduction in carotenoid bioaccessibility may be attributed to dietary fiber, as fiber inhibits carotenoid micellization in the intestine (HadiNezhad & Butler, 2009).

**Table 4:** Bioaccessibility of polyphenols and carotenoids of CMS-supplemented cookies.

Substances	Bioaccessibility	Units	Control	CMS-supplemented cookies				
				5 %	10 %	15 %	20 %	25 %
Polyphenols	Released content	mg GAE/100 g db	9.57 ± 0.39ᵃ	15.54 ± 0.36ᵇ	20.51 ± 0.56ᶜ	25.44 ± 0.85ᵈ	37.63 ± 0.20ᵉ	45.27 ± 1.02ᶠ
	Bioaccessibility	%	35.80 ± 1.47ᵃ	43.08 ± 1.00ᵇ	46.17 ± 1.25ᶜ	51.36 ± 1.71ᵈ	55.17 ± 0.29ᵉ	61.50 ± 1.39ᶠ
Carotenoids	Released content	μg/100 g db	5.41 ± 0.41ᵃ	72.40 ± 0.77ᵇ	116.74 ± 4.10ᶜ	153.56 ± 2.44ᵈ	200.12 ± 7.93ᵉ	216.83 ± 4.48ᶠ
	Bioaccessibility	%	18.95 ± 1.43ᵃ	16.36 ± 0.17ᵇ	14.11 ± 0.49ᶜ	12.29 ± 0.20ᵈ	11.45 ± 0.45ᵉ	10.65 ± 0.22ᶠ

Data are presented as mean ± standard deviation (n = 3). Means with different superscript letters in each row indicate significant differences (p < 0.05); Abbreviations: CT: control cookies without CMS supplementaton; 5 %, 10 %, 15 %, 15 %, 20 %, 25 %: cookies supplemented with 5 %, 10 %, 15 %, 15 %, 20 %, and 25 % CMS; CMS: *C. militaris* substrate; GAE: gallic acid equivalent.

## 4 Conclusion

In conclusion, CMS-supplemented cookies demonstrated both nutritional and functional benefits, offering a promising option for healthy food products. Dietary fibers, polyphenols, and carotenoids elevated 1.41–2.95 times, 1.30–2.62 times, and 15.50–71.34 times, respectively. In addition, the free radical scavenging activity increased 4.14–14.03 times, while metal reducing power enhanced 2.17–7.39 times. Although CMS incorporation induced physicochemical changes, it did not significantly affect sensory evaluation at supplementation levels of 5–15 %. Furthermore, CMS supplementation lowered the GI of cookies, classifying them within the low-GI food group, while promoting the intestinal release of polyphenols and carotenoids 1.62–4.7 times and 13.38–40.07 times. These findings highlight the potential of CMS powder as a functional ingredient for food innovation and sustainability. Future research should further investigate the effects of CMS supplementation on the nutritional and physicochemical properties of cookies during storage.

## Use of AI tools declaration

The authors declare they have not used artificial intelligence (AI) tools in the creation of this article.

## CRediT authorship contribution statement

**Chanh M. Nguyen:** Writing – original draft, Visualization, Investigation, Funding acquisition, Formal analysis, Data curation. **Khoa D. Nguyen:** Writing – review & editing, Writing – original draft, Visualization, Formal analysis, Data curation. **Truc N.T. Tran:** Investigation, Formal analysis. **Tin H. Trang:** Investigation, Formal analysis. **N.M.N. Ton:** Conceptualization, Methodology, Project administration, Resources, Supervision, Validation. **V.V.M. Le:** Conceptualization, Methodology, Project administration, Resources, Supervision, Validation. **T.T.T. Tran:** Writing – review & editing, Validation, Supervision, Resources, Project administration, Methodology, Conceptualization.

## Declaration of competing interest

There are no competing financial interests or personal relationships to influence the work reported in the present study.

The authors declare that they have no known competing financial interests or personal relationships that could have appeared to influence the work reported in this paper.

## Data Availability

Data will be made available on request.

## Appendix A Supplementary data

Supplementary material **Fatty acid profile of control cookies (0% supplement CMS) and CMS-supplemented cookies (15% supplement)** Image 1
