# Supplementary material for: Low-glycemic index cookies supplemented with Cordyceps militaris substrate: Nutritional values, physicochemical properties, antioxidant activity, bioactive constituents, and bioaccessibility
Source: Food Chem X. 2025 Apr 23;27:102494. doi: 10.1016/j.fochx.2025.102494 (PMC12088761; doi:10.1016/j.fochx.2025.102494)
Supplement: Supplementary file 1 — Supplementary material Fatty acid profile of control cookies (0% supplement CMS) and CMS-supplemented cookies (15% supplement) [file mmc1.docx]

Low-Glycemic Index Cookies Supplemented with *Cordyceps militaris* Substrate: Nutritional Values, Physicochemical Properties, Antioxidant Activity, Bioactive Constituents, and Bioaccessibility

**Chanh M. Nguyen^1,2^, Khoa D. Nguyen^2,3^, Truc N.T. Tran^1,2^, Tin H. Trang^1,2^, N.M.N. Ton^1,2^, V.V.M. Le^1,2^, T.T.T. Tran^1,2*^**

^1^ Department of Food Technology, Ho Chi Minh City University of Technology (HCMUT), Ho Chi Minh City, Vietnam

^2^ Vietnam National University – Ho Chi Minh City (VNU-HCM), Ho Chi Minh City, Vietnam

^3^ Department of Biochemistry, Faculty of Biology—Biotechnology, University of Science, Ho Chi Minh City, Vietnam

**Correspondence:**

**T.T.T. Tran**

Email: ttttra@hcmut.edu.vn Tel. (+ 84)903377630

# Conflict of Interest Statement: The authors declare no conflicts of interest.

# Type of article: Research article

# Table S1

# Fatty acid profile of control cookies (0% supplement CMS) and CMS-supplemented cookies (15% supplement)

| **Fatty acid profile** | | **Content (%)** | |
| --- | --- | --- | --- |
|  |  | **Control** | **CMS-15%** |
| C8:0 | Acid octanoic | 0.170 | 0.158 |
| C10:0 | Acid capric | 0.130 | 0.124 |
| C12:0 | Acid lauric | **1.460** | **1.394** |
| C14:0 | Acid myristic | 0.627 | 0.613 |
| C16:0 | Acid palmitic | **8.880** | **8.670** |
| C16:1 | Acid palmitoleic | 0.074 | 0.066 |
| C17:0 | Acid margaric | 0.025 | 0.021 |
| C18:0 | Acid stearic | 1.270 | 1.245 |
| C18:1 | trans acid elaidic | 0.008 | 0.009 |
| C18:1 | Acid cis-oleic | **7.970** | **7.650** |
| C18:2 | Acid cis-linoleic | **2.760** | **2.620** |
| C18:3 | Acid alpha-linolenic | 0.107 | 0.096 |
| C20:0 | Acid arachidic | 0.081 | 0.077 |
| C20:1 | Acid eicosenoic | 0.037 | 0.036 |
| C20:4 | Acid arachidonic | 0.022 | 0.000 |
| C22:0 | Acid behenic | 0.050 | 0.054 |
| C22:6 | DHA | 0.011 | 0.009 |
| C24:0 | Acid lignoceric | 0.019 | 0.023 |
| Saturated fatty acids (SFA) | | 12.70 | 12.40 |
| Unsaturated fatty acids (UFA) | | 11.00 | 10.50 |
| Monounsaturated fatty acid (MUFA) | | 8.10 | 7.76 |
| Polyunsaturated fatty acid (PUFA) | | 2.90 | 2.72 |
